# Pax3 inhibits Neuro‐2a cells proliferation and neurite outgrowth

**DOI:** 10.1111/jcmm.16195

**Published:** 2020-12-17

**Authors:** Bingqing Huo, Yang Yang, Manhui Li, Jun Wan, Wei Zhang, Bo Yu, Xiaofan Chen

**Affiliations:** ^1^ Biomedical Research Institute Shenzhen Peking University ‐ The Hong Kong University of Science and Technology Medical Center Shenzhen China; ^2^ Greater Bay Biomedical Innocenter Shenzhen Bay Laboratory Shenzhen China; ^3^ Department of Dermatology Peking University Shenzhen Hospital Shenzhen China

**Keywords:** Cell cycle, Neurite outgrowth, Neuro‐2a cells, Pax3, Pax7, Proliferation

## Abstract

Pax3 and Pax7 are closely related transcription factors that are widely expressed in the developing nervous system and somites. During the normal development in the central nervous system (CNS), Pax3 and Pax7 are mainly expressed in the dorsal part of the neural tube. Further analysis revealed that Pax3 and Pax7 shared redundant functions in the spinal cord development. However, it is still unknown whether Pax3 and Pax7 play a role in neuronal differentiation. In this study, Pax3 and Pax7 genes were overexpressed in Neuro‐2a, the mouse neuroblastoma cell line. CCK‐8 and EdU assay results showed that overexpression of Pax3 inhibited cell viability and proliferation of Neuro‐2a cells, whereas the overexpression of Pax7 had no significant difference on their cell viability and proliferation. Overexpression of Pax3 not only increased the percentage of cells in the S phase and G0/G1 phase, but also decreased that in the G2 phase. Moreover, the total neurite lengths of Neuro‐2a cells were significantly shorter in Pax3 overexpressed group than those in negative control group and showed no significant difference between Pax7 overexpressed group and negative control group. These results suggested that Pax3 not only inhibited the cell viability and proliferation but also affected the cell cycle and the neurite outgrowth of Neuro‐2a cells. RNA sequencing analysis showed up‐regulated genes in Pax3 overexpressed group were involved in cell cycle machinery, which may reveal the potential mechanism of Neuro‐2a cells proliferation.

## 1 INTRODUCTION

The Paired box (Pax) genes encode transcription factors which participate in the induction of neural crest and the formation of neural crest‐derived structures.^1^ Pax3 and its paralogs Pax7 were derived from an ancestral Pax3/7 gene during the evolution of vertebrate. They share highly homologous sequence and show partially overlapping expression pattern during embryogenesis.^2^, ^3^, ^4^, ^5^ In some neural crest lineages, such as sensory neurons, Schwann cells and melanoblast, Pax3 and Pax7 were expressed during the proliferation of their progenitors.^6^, ^7^, ^8^, ^9^, ^10^ Both genes are essential to regulate the closure of neural tube and the programme of post‐mitotic differentiation.^11^

In mouse, the expression of Pax3 exists at gestational day 8, whereas that of Pax7 appears at gestational day 9..^12^As reported, mice with homozygous null Pax3 mutations are featured as embryonic lethal at the late embryonic stage. The *splotch* mutant mouse, which bear mutations within the Pax3 gene, exhibited neural crest‐derived defects.^13^, ^14^ It suggested Pax3 played the essential role in neural crest development.^15^, ^16^ Moreover, Pax3 played vital roles in the survival of cardiac neural crest cell during neural crest cell migration.^17^ Pax7 contributes to the differentiation of neural cells from neural precursors. In vitro, embryonic teratocarcinoma mouse cells can be differentiated into neural cells through Pax7 induction.^18^ However, pax7 mutant mice show no obvious neural crest phenotype in the CNS. As further analysis revealed, Pax3 functions partially overlapped with Pax7 during the development of spinal cord.^19^, ^20^, ^21^

The function of Pax3 and Pax7 during neural crest development has been well studied. However it is still unknown whether Pax3 and Pax7 play a role in neural cells proliferation and neurite outgrowth. Neuro‐2a cells are more effective and economical than primary neuronal cultures, which was chosen for studies about neuronal survival, neuronal differentiation and so on.^22^, ^23^, ^24^, ^25^ Therefore, in this study, we elucidated the function of Pax3 and Pax7 in postnatal neuronal through Neuro‐2a cell line. Our results suggested that Pax3 not only inhibited the cell viability and proliferation but also affected the cell cycle and the neurite outgrowth of Neuro‐2a cells.

## 2 MATERIALS AND METHODS

### 2.1 Recombinant adenoviral vector construction

LR Clonase Ⅱ Enzyme Mix (Invitrogen, USA) was used to generate an expression clone, which contains the Pax3 and Pax7 gene and ZsGreen1. After digested by PacI (New England Biolabs, USA), the expression clone was introduced into 293A cells by Lipofectamine 2000 (Invitrogen, USA). Adenovirus‐containing cells and media could be harvested by squirting cells off the plate when approximately 80% cytopathic effect is observed (typically 7‐10 days after transfection). The crude viral lysate is prepared by using several freeze/thaw cycles followed by centrifugation. Finally, we obtained adenoviral‐Pax3, adenoviral‐Pax7 and adenoviral‐Negative Control (mark as Ad‐Pax3, Ad‐Pax7 and Ad‐NC).

### 2.2 Cell culture and differentiation conditions

Mouse neuroblastoma Neuro‐2a cells (ATCC, CCL‐131) were cultured in Dulbecco's modified Eagle's medium (DMEM, Gibco Inc, USA) supplied with 100U/mL penicillin, 100mg/mL streptomycin and 10% foetal bovine serum (FBS, Gibco, USA). Cells were cultured at 37 ºC in cell culture flasks with 5% CO_2_. In vitro differentiation studies, 1 × 10^4^ Neuro‐2a cells were plated in 12‐well plated for 24 hours. The medium was replaced by DMEM with 1% foetal bovine serum and retinoic acid at a dilution of 1:1000. The medium was replaced every 3 days.

### 2.3 RNA extraction and quantitative real‐time PCR (qRT‐PCR) assay

Total RNA was extracted from Neuro‐2a cells according to TRIzol protocol. Then, 2000 ng of total RNA was reversely transcripted with GoScript™ (Promega, USA) following the manufacturer instructions. GAPDH was used as an internal control to normalize Pax3 and Pax7 expression. Real‐time PCR was carried out with SYBR Green Super mix (Bio‐RAD, USA). The 2^‐ΔΔCT^ method is used to calculate the relative expression.

Pax3 primer sequence: forward: 5’‐AAGCCCAAGCAGGTGACAA‐3’, reverse: 5’‐ATGGAACTCACTGACGGCAC‐3’. Pax7 primer sequence: forward: 5’‐GCTACCAGTACAGCCAATATG‐3’, reverse: 5’‐GTCACTAAGCATGGGTAGATG‐3’. GAPDH primer sequence: forward: 5’‐AACTTTGGCATTGTGGAAGG‐3’, reverse: 5’‐ATGCAGGGATGATGTTCT‐3’.

### 2.4 Western blot analysis

Neuro‐2a cells were collected and washed with cold PBS, and then protein lysis buffer (RIPA buffer) was added to the culture plate. The adherent cells were scraped down. The cell suspension was gently transferred into an ice‐cold centrifuge tube and centrifuged at 4 ºC and 12 000 rpm for 15 min. The supernatant was transferred to a new ice‐cold centrifuge tube, and the cell debris was discarded. The concentration of each protein sample was determined by a BCA kit (Beyotime, China). Then, 40 µg of total protein was determined with 10% SDS‐PAGE. Targeted proteins were transferred onto PVDF (polyvinylidene difluoride) membrane. After blocked with 5% low‐fat dried milk for 2h at room temperature, membranes were incubated with corresponding primary antibodies(Cell Signaling Technology, USA) at a dilution of 1:1000 overnight at 4ºC. After washed with PBST three times, membranes were incubated with HRP (horseradish peroxidase) labelled sheep anti‐rabbit secondary antibody (Sigma‐Aldrich, USA) at a dilution of 1:10 000 at room temperature for 1h. Finally, signals were detected with enhance chemiluminescent reagents.

### 2.5 EdU assay

The proliferation of Neuro‐2a cells was determined by 5‐ethynyl‐2’‐deoxyuridine (EdU) assay. After 48h infected by Ad‐Pax3, Ad‐Pax7 and Ad‐NC, EdU (Ribobio, China) were added in order to incorporate into the DNA of proliferating Neuro‐2a cells. EdU was added to the culture medium for 1 hour in order to incorporate into the replicating DNA. Then, cultured cells were washed three times with PBS and fixed with 4% paraformaldehyde for 20 min. Staining was performed with Cell‐Light^TM^ EdU Apollo®567 In Vitro Kit (Ribobio, China) according to the instructions. Subsequently, the DNA contents of the cells were stained with Hoechst 33 342 (Beyotime, China) for 30min. Finally, EdU‐labelled cells were counted with fluorescent microscope and normalized by the total number of Hoechst‐stained cells.

### 2.6 CCK‐8 assay

Neuro‐2a cells were seeded into 96‐well plates (about 1000 cells per well) overnight. The infection time‐point was marked as 0h. 10 µL Cell Counting Kit‐8 solution (MedChem Express, USA) was added into each well of 96‐well plates at 0, 12, 24, 36, 48, 60, 72, 84 and 96h. The cells were incubated for 1h at 37 ºC incubator with 5% CO_2_. Then, the absorbance at the wavelength of 450nm was determined to measure cell proliferation.

### 2.7 Cell cycle progression analysis

To determine the distribution of the cells in each phase of the cell cycle, the Neuro‐2a cells were seeded into 10 cm dish and then cultured in DMEM containing 10% FBS at 37 ºC for 24h. After infected by adenoviral for 48h, the cells were collected and washed with PBS, fixed with 70% ethanol for about 4h, and then washed again with PBS. The cells were re‐suspended in a solution of propidium iodide (50 µL/mL) and RNaseI (250 µg/mL), followed by incubation for 30 min at room temperature. Analysis of cell cycle was carried out with flow cytometry. The proportions of cells in G0/G1, S and G2/M phases were determined with the computer program FlowJo software (Becton, Dickinson & Company, USA).

### 2.8 Neurite outgrowth analysis

Fluorescent microscope was used to carry on morphological analysis and quantify the length of neurite. At least ten fields were picked out randomly, of which over 100 cells were selected for the following analysis of the neurite outgrowth of Neuro‐2a cells. Neuro‐2a cells with neurite equal to or longer than their cell body were defined as positive neurite outgrowth.^26^ The neurite length was identified and defined as the total neurite number and length per cell per field. Image J software was applied in the analysis of neurite outgrowth of Neuro‐2a cells.

### 2.9 Immunofluorescence

The expression of MAP2 in Neuro‐2a cells was examined by immunofluorescence assay. Neuro‐2a cells were infected with Ad‐Pax3, Ad‐Pax7 and Ad‐NC, respectively. After cultured for 48 hours, the cells were washed with PBS three times. It was fixed with 4% paraformaldehyde for 30 min at room temperature. After washed three times with PBS, the cells were blocked in blocking buffer (SL038, Solarbio, China) for 1h and incubated with 1:50 dilution of anti‐MAP2 antibody (1:50, 4544S, Cell Signaling Technology, USA) overnight at 4 ºC. After incubation with the primary antibody, the cells were washed three times with PBS and incubated with a corresponding secondary antibody for 1h. The secondary antibodies were Cy3‐labelled goat anti‐rabbit‐IgG antibody (1:10 000, Sigma, USA). After further washed three times with PBS, cell nuclei were stained with DAPI for 3 min at room temperature. After washed with PBS, cells were observed and images were acquired with fluorescent microscope.

### 2.10 Library preparation and RNA sequencing

Neuro‐2a cells were infected with Ad‐Pax3 for 48h. Total RNA was extracted from Neuro‐2a cells according to TRIzol protocol. The quality and quantity of RNA samples (n = 2 per group) were checked with NanoPhotometer spectrophotometer (IMPLEN, USA). The FPKMs values of transcripts were calculated by using Cuffdiff (v. 2. 1. 1) to evaluate the expression levels of protein‐coding genes in each sample.^27^ Transcripts with p values less than 0.05 were regarded as being differentially expressed. Normalized expression= (mapped reads)/ (total reads) x1, 000, 000.^28^

Gene Ontology (GO) analysis designed by the Gene Ontology Consortium was performed to figure out significant functions of target genes.^29^ Kyoto Encyclopedia of Genes and Genomes (KEGG) was used for pathways analysis of target genes.^30^ GO and KEGG terms featuring p values less than 0. 05 were considered significantly enriched.

### 2.11 Statistical analysis

All data are shown as mean and SEM. Statistical analysis was performed by Prism (GraphPad Software, San Diego, CA), Statistical Product and Service Solutions (SPSS19. 0, IBM Corp. , USA) to determine the significance of differences between the treated group and the control group. Classification data were measured by t test. *P* < .05 was considered as statistically significant.

### 2.12 Data access

All raw and processed sequencing data have been submitted to the NCBI Gene Expression.

Omnibus (GEO; http://www.ncbi.nlm.nih.gov/geo/) under accession number GSE146058.

## 3 RESULTS

### 3.1 Expression changes of Pax3 and Pax7 after Neuro‐2a cells infected by Ad‐Pax3 and Ad‐Pax7

Neuro‐2a is a mouse neural crest‐derived cell line that has been extensively used to study neuronal differentiation, neurite growth and signalling pathways.^31^ Retinoic acids (RA) can induce differentiation of these cells into neurons within a few days.^32^ The PAX3 isoforms in human are designated as PAX3a, PAX3b, PAX3c, PAX3d, PAX3e, PAX3g and PAX3h.^33^ However, there are only two isoforms of PAX3 transcripts in mouse, Pax3‐201 (ENSMUST00000004994.15) and Pax3‐202 (ENSMUST00000087086.6). Compared with 7 human PAX3 amino acid sequence, Pax3‐201 has the highest homology with PAX3d (up to 98.76%), whereas Pax3‐202 has the highest homology with PAX3c (up to 98.74%) (Figure S1). Only 5 amino acids at the extreme carboxy termini differed Pax3‐201 from Pax3‐202. Pax3‐201 was chosen to analysis the Pax3 gene functions in the Neuro‐2a cells, because it had been annotated by APPRIS, which is a system to identify the most functionally important transcript. The expression of Pax3 was increased gradually during RA‐induced neurite outgrowth (Figure S2). However, the expression level of Pax7 keeps so low that no significant change of Pax7 expression was detected during RA‐induced neurite outgrowth.

**FIGURE 1:**
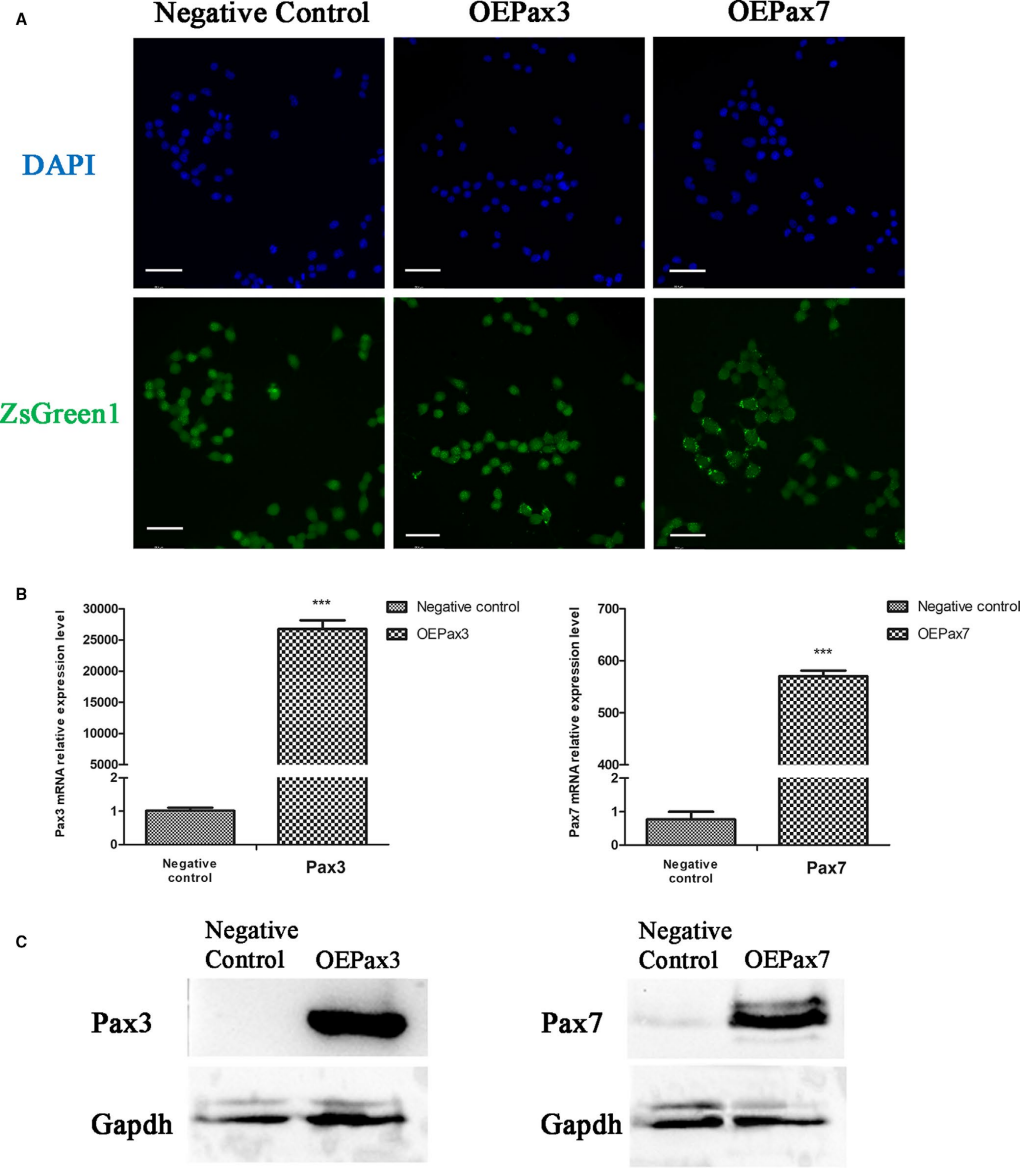
Neuro‐2a cells were infected by Ad‐Pax3 and Ad‐Pax7. A, Fluorescence of Neuro‐2a cells infected with Ad‐Pax3 and Ad‐Pax7 for 48h. Scale bars = 50 μm. B, The mRNA expression levels of Pax3 and Pax7 in Neuro‐2a cells infected by Ad‐Pax3 and Ad‐Pax7 were significantly increased for 48h. C, The protein expression levels of Pax3 and Pax7 in Neuro‐2a cells infected by Ad‐Pax3 and Ad‐Pax7 were significantly increased for 48h. The data represent the means ± SEM, n = 3 independent experiments. ***P < .001 vs. Negative control

Pax3 and Pax7 genes were inserted into an adenovirus vector and packaged into an adenovirus to infect Neuro‐2a cells for overexpression. After infection, we obtained two groups, overexpressing Pax3 and Pax7 (mark as OEPax3 and OEPax7). As shown in Figure 1A, strong expression of ZsGreen1 indicated that the recombinant adenovirus‐containing Pax3 and Pax7 gene respectively were successfully constructed. Real‐time PCR results revealed that the expression levels of Pax3 and Pax7 mRNA were significantly increased after Neuro‐2a cells were infected by Ad‐Pax3 and Ad‐Pax7 (Figure 1B). Western blot analysis also revealed that the protein levels of Pax3 and Pax7 expressed in Neuro‐2a cells were significantly up‐regulated in Neuro‐2a cells infected by Ad‐Pax3 and Ad‐Pax7 (Figure 1C).

### 3.2 Pax3 inhibited Neuro‐2a cells viability

**FIGURE 2:**
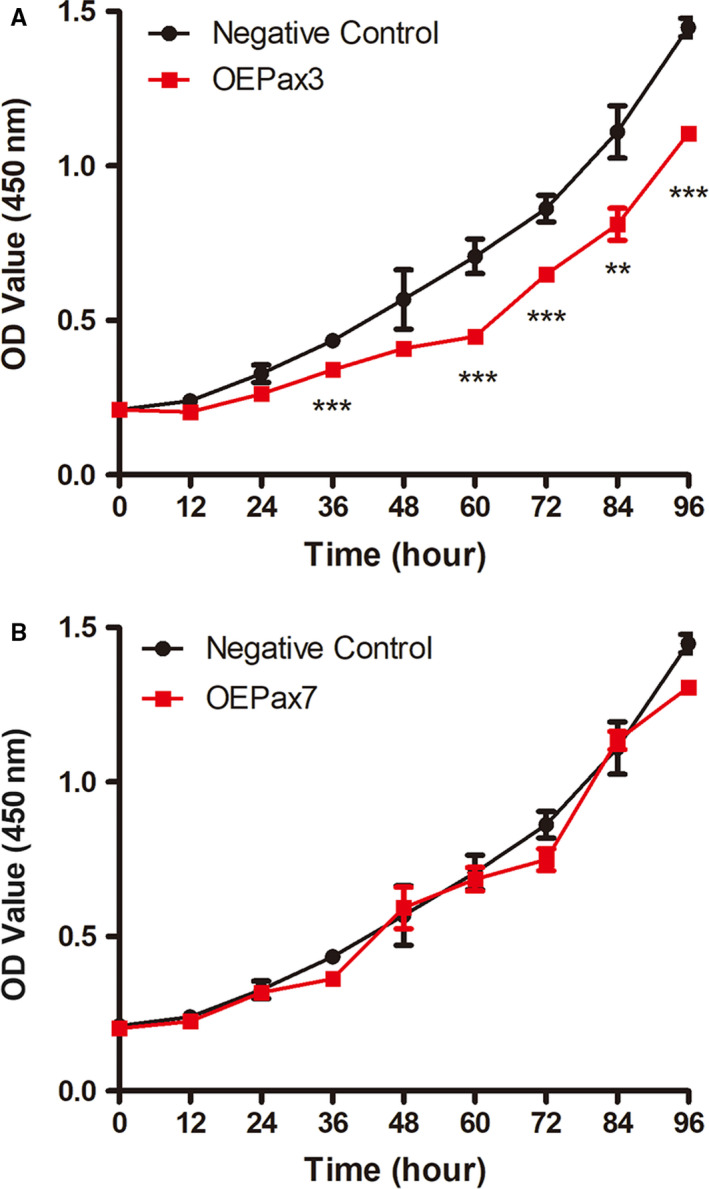
The viability of Neuro‐2a cells overexpressing Pax protein. A, The cell viability was significant decrease in Neuro‐2a cells infected by Ad‐Pax3 at 36, 60, 72, 84 and 96h. B, There was no significant change in the cell viability after Neuro‐2a cells infected by Ad‐Pax7. The data represent the means ± SEM, n = 3 independent experiments. **P < .01, ***P < .001 vs. Negative control

In previous studies, Ad‐Pax3 and Ad‐Pax7 have been found to effectively increase the expression of Pax3 and Pax7 in Neuro‐2a cells, respectively. As a result, the viability of Neuro‐2a cells overexpressing Pax3 and Pax7 was investigated by the CCK‐8 assay. As shown in Figure 2A, A significant decrease in cell viability of the Pax3 overexpression group was observed at 36h, 60h, 72h, 84h and 96h compared with the negative control group. As shown in Figure S3, cells transfected by 1:2, 1:20 and 1:200 diluted Ad‐Pax3 virus showed similar phenotypes as that infected with undiluted Ad‐Pax3 virus, which suggested proper induction of the PAX3 expression can also inhibit the viability of Neuro‐2a cells. However, the Pax7 overexpression group has no significant differences (Figure 2B). Taken together, it suggested that Pax3 can inhibit the cell viability of Neuro‐2a cells.

### 3.3 Pax3 inhibited Neuro‐2a cells proliferation

**FIGURE 3:**
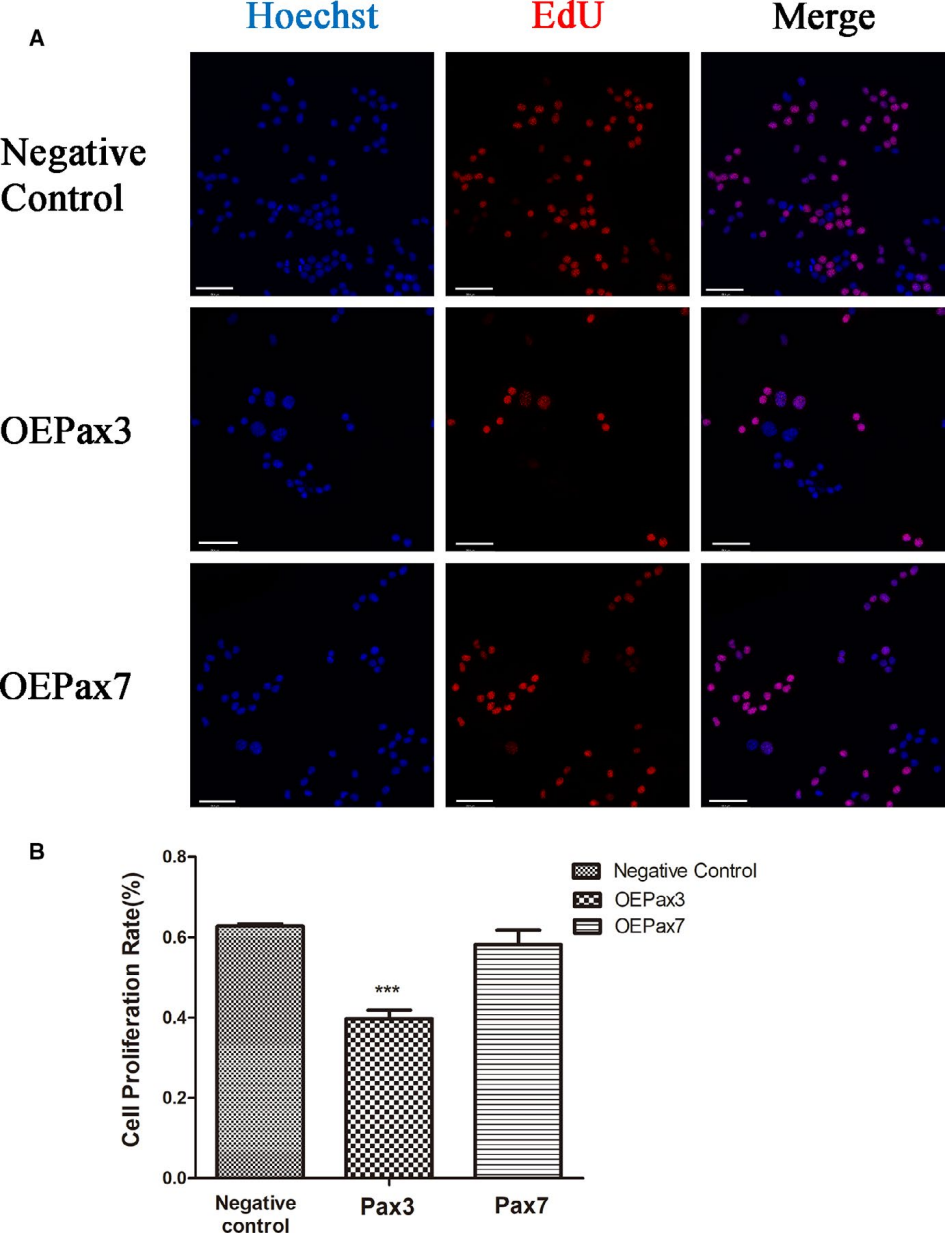
Inhibitory effect of Pax3 on Neuro‐2a cells. A, EdU fluorescence staining was used to detect the newly synthesized DNA. Scale bars = 50 μm. B, The percentage of EdU positive cells was calculated by Image J software. The cell proliferation was significant decreased in Neuro‐2a cells after Ad‐Pax3 infection. Cell proliferation has no significant change after Ad‐Pax7 infection. The data represent the means ± SEM, n = 3 independent experiments. ***P < .001 vs. Negative control

The effect of overexpression of Pax3 and Pax7 on the proliferation of Neuro‐2a cells was investigated by the EdU assay. As shown in Figure 3A, EdU‐labelled cells can be counted with fluorescent microscope. Ad‐Pax3 Infection for 48h resulted in a significant decreased number of proliferating Neuro‐2a cells. Similarly, the EdU assay showed the same inhibitory pattern on Neuro‐2a cells proliferation. However, the Pax7 group was not significantly influenced after infected by Ad‐Pax7 compared with the negative control group (Figure 3B). These results indicated that Pax3 played an inhibitory effect on Neuro‐2a cells proliferation.

### 3.4 The effect of Pax3 and Pax7 on Neuro‐2a cell cycle progression

**FIGURE 4:**
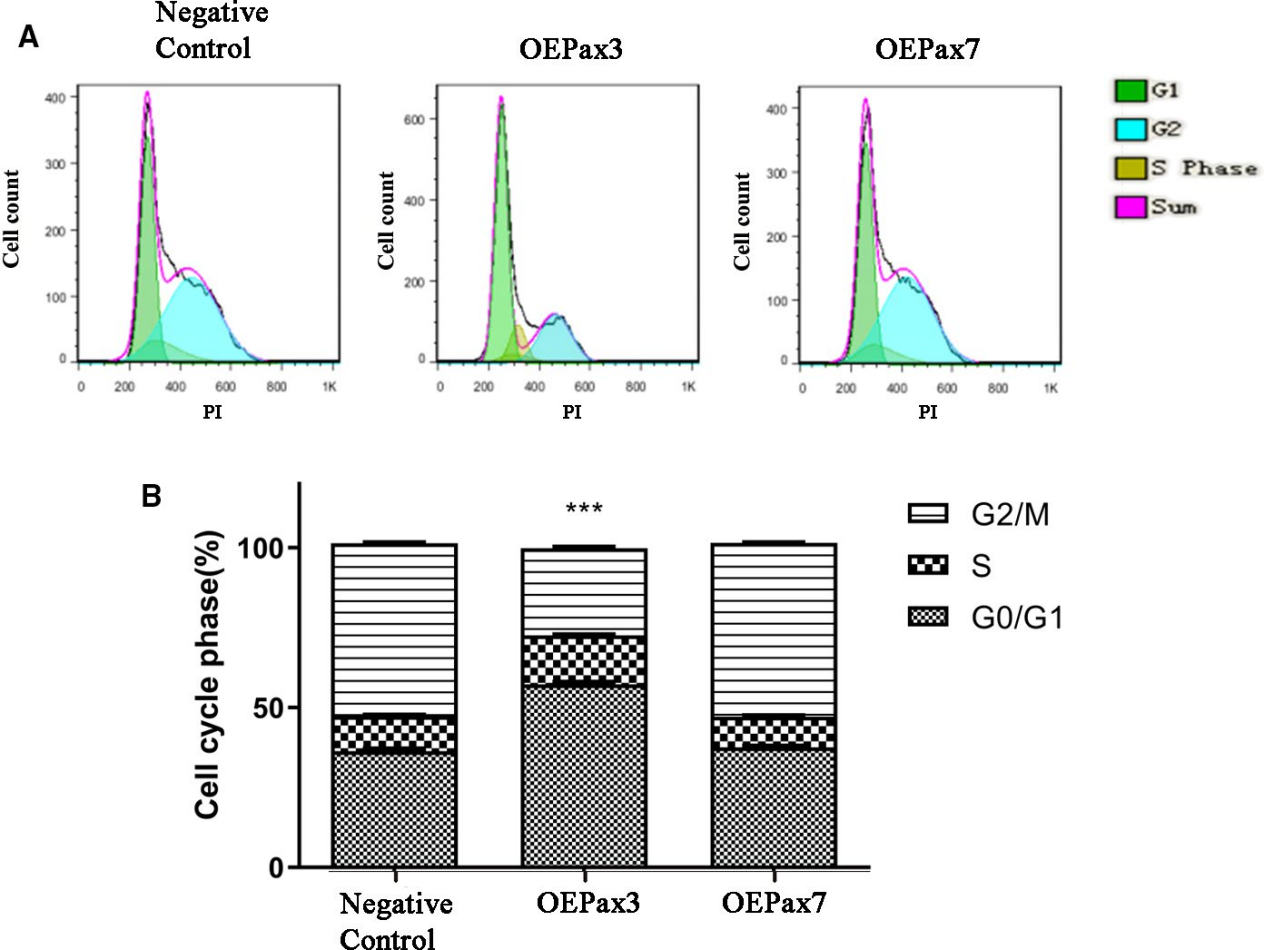
The effect of Pax3 and Pax7 on Neuro‐2a cell cycle progression. Cell cycle progression of Neuro‐2a cells infected by Ad‐Pax3, Ad‐Pax7 and Ad‐NC for 48h was observed with flow cytometry analysis. A, Individual nuclear DNA content was reflected by the fluorescence intensity of incorporated propidium iodide. B, The percentage of cell population at each stage. Overexpression of Pax3 may induce S phase arrest in Neuro‐2a cells. Each item is derived from representative experiments, and the data were obtained from at least 10, 000 events. The data represent the means ± SEM, n = 3 independent experiments. ***P < .001 vs. Negative control

We determined whether Pax3 and Pax7 may modulate the cell cycle progression of Neuro‐2a cells with flow cytometry analysis. Compared with that in the control group, the percentage of the cells in the S phase and G0/G1 phase were increased, whereas the percentage of the cells in the G2 phase were decreased in the Pax3 overexpressed group. However, the Pax7 overexpressed group still displayed no significantly difference. Taken together, it indicated that Pax3 may induce S phase arrest in Neuro‐2a cells (Figure 4).

### 3.5 Pax3 may inhibit neurite outgrowth of differentiated Neuro‐2a cells

**FIGURE 5:**
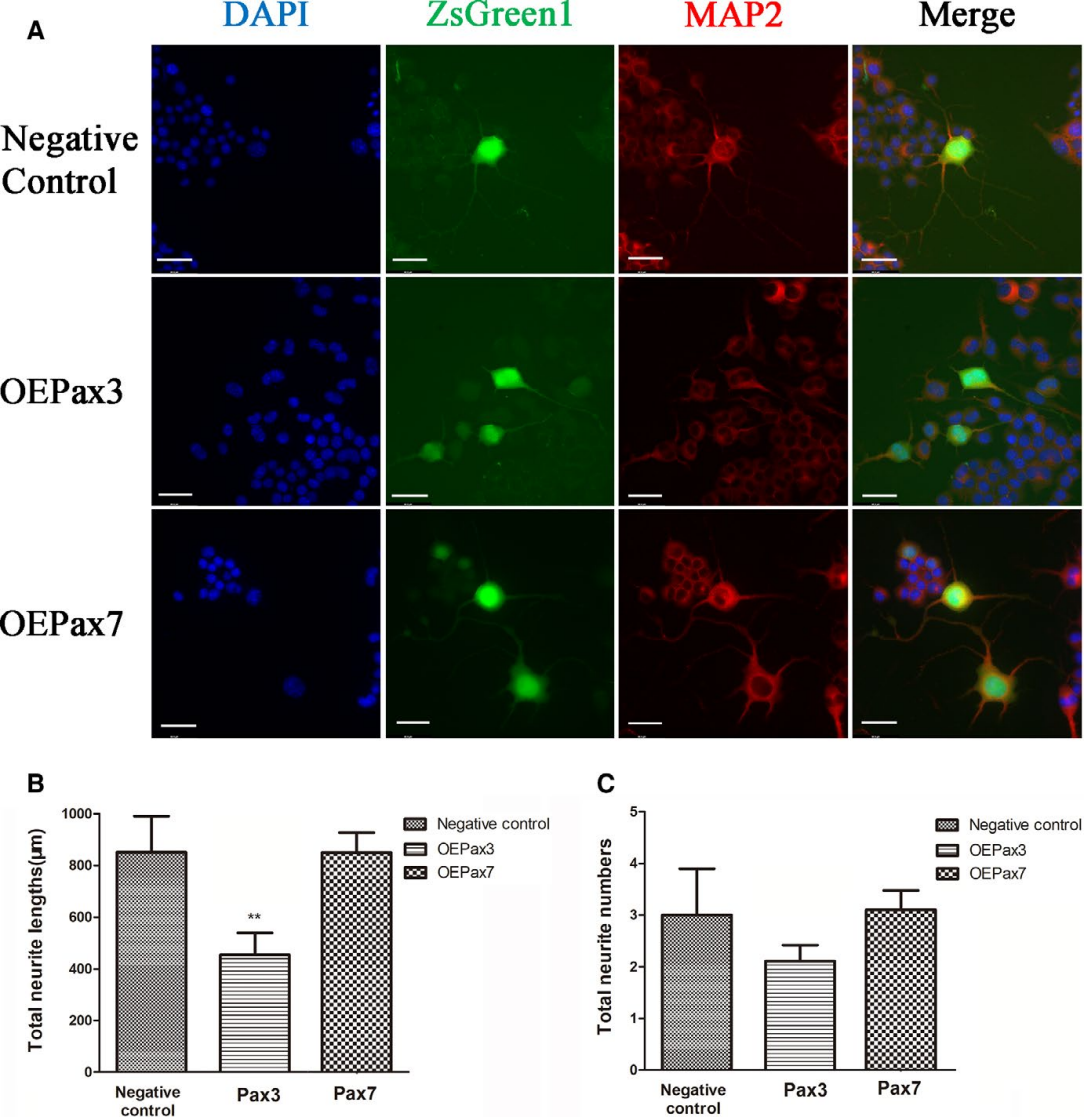
The effect of Pax3 and Pax7 on neurite outgrowth of differentiated Neuro‐2a cells. A, Cell immunofluorescence images of all nuclei (DAPI), MAP2 and ZsGreen1 among Pax3 overexpressed group, Pax7 overexpressed group and negative control group. Scale bar = 50µm. B, Total neurite lengths of positive cells in Pax3 overexpressed group were significant shorter than those in the negative control. Pax7 overexpressed group displayed no significant difference. Negative control: 851.4 ± 313. 1µm, Pax3 overexpressed group: 454.3 ± 226.4µm, Pax7 overexpressed group: 850 ± 216.6µm. **P < .01 vs. Negative control. C, Pax3 overexpressed group and Pax7 overexpressed group displayed no statistical difference of total neurite numbers compared with the negative control group in total neurite numbers. The data represent the means ± SEM, n = 3 independent experiments

To determine the biological effect of Pax3 and Pax7 on neurite outgrowth, we performed functional analysis with Neuro‐2a cells infected by Ad‐Pax3, Ad‐Pax7 and Ad‐NC. Cells were differentiated after treated with retinoic acid according to the instruction. MAP2, a marker of neuron, was used to label the differentiated Neuro‐2a cells (Figure 5A). Cells expressing both ZsGreen1 and MAP2 were identified as the positive cells.

Cell immunofluorescence results showed that cells expressing both ZsGreen1 and MAP2 in Pax3 overexpressed group exhibited significantly decreased total neurite lengths, whereas that of Pax7 overexpressed group displayed no significant difference (Figure 5B). Total neurite numbers were examined among the OEPax3, OEPax7 and negative control group. As was showed in Figure 5C, Pax3 overexpressed group and Pax7 overexpressed group displayed no statistical difference compared with the negative control group. Overall, it suggested that Pax3 may inhibit neurite outgrowth of differentiated Neuro‐2a cells.

### 3.6 Transcriptome analysis of Neuro‐2a cells overexpressing Pax3

To investigate the underlying mechanisms of inhibitory of Cell viability, proliferation and cell cycle by Pax3, we performed RNA sequencing in Pax3 overexpressed group and negative control group. 25 910 transcripts with significant P value included 1047 up‐regulated genes, 1313 down‐regulated and 23 550 unchanged transcripts.

**FIGURE 6:**
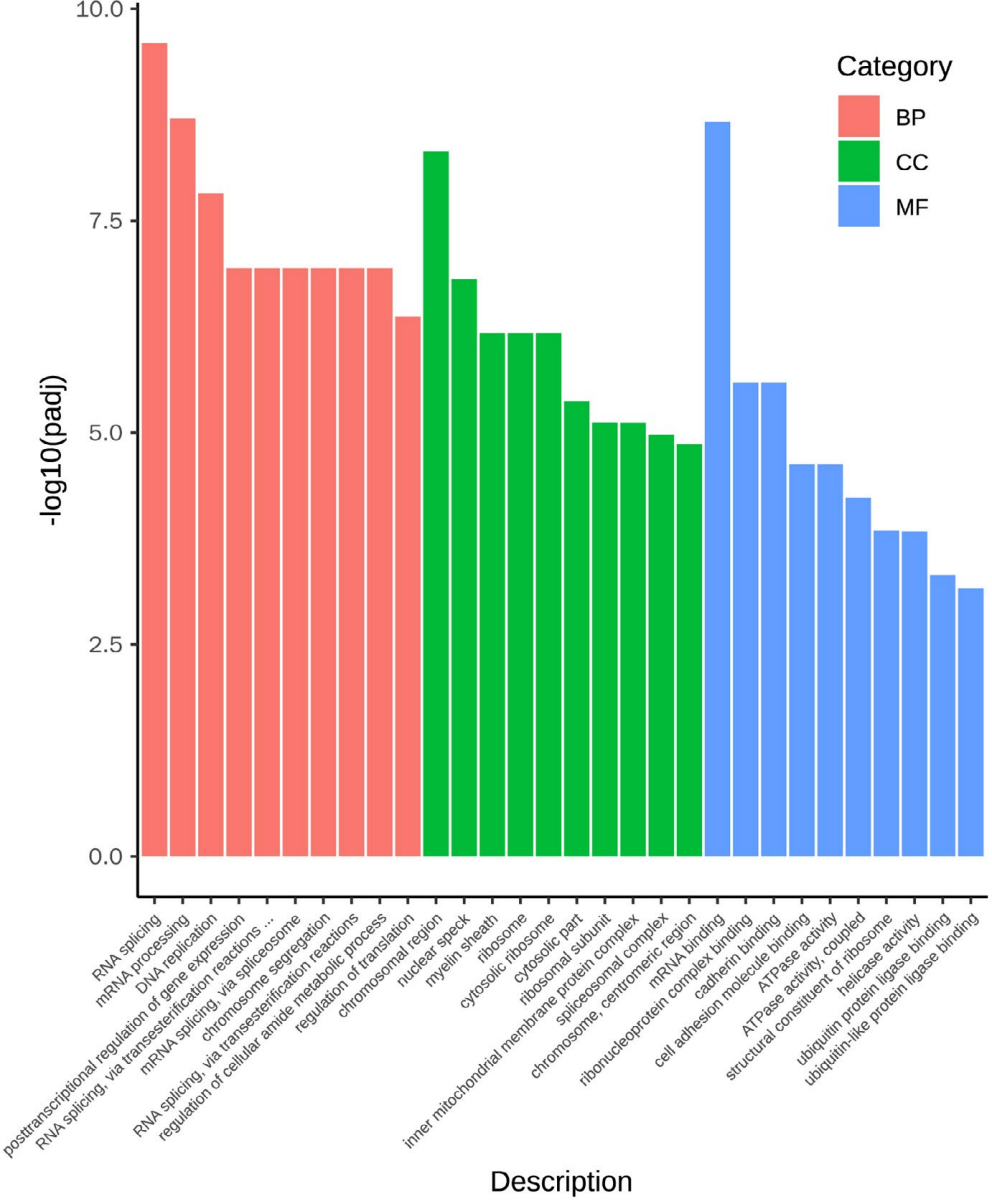
GO analysis of Neuro‐2a cells infected by Ad‐Pax3. Compared with the negative control group, GO terms were enriched under biological processes (red columns), cellular component (green columns) and molecular function (blue columns) category in Pax3 overexpressed group

P‐Significant transcripts were analysed for enriched GO terms. Ten GO terms were enriched under biological processes, cellular component and molecular function category, respectively. The most significant GO biological process was 'RNA splicing' (GO: 0 008 380) (Figure 6).

**FIGURE 7:**
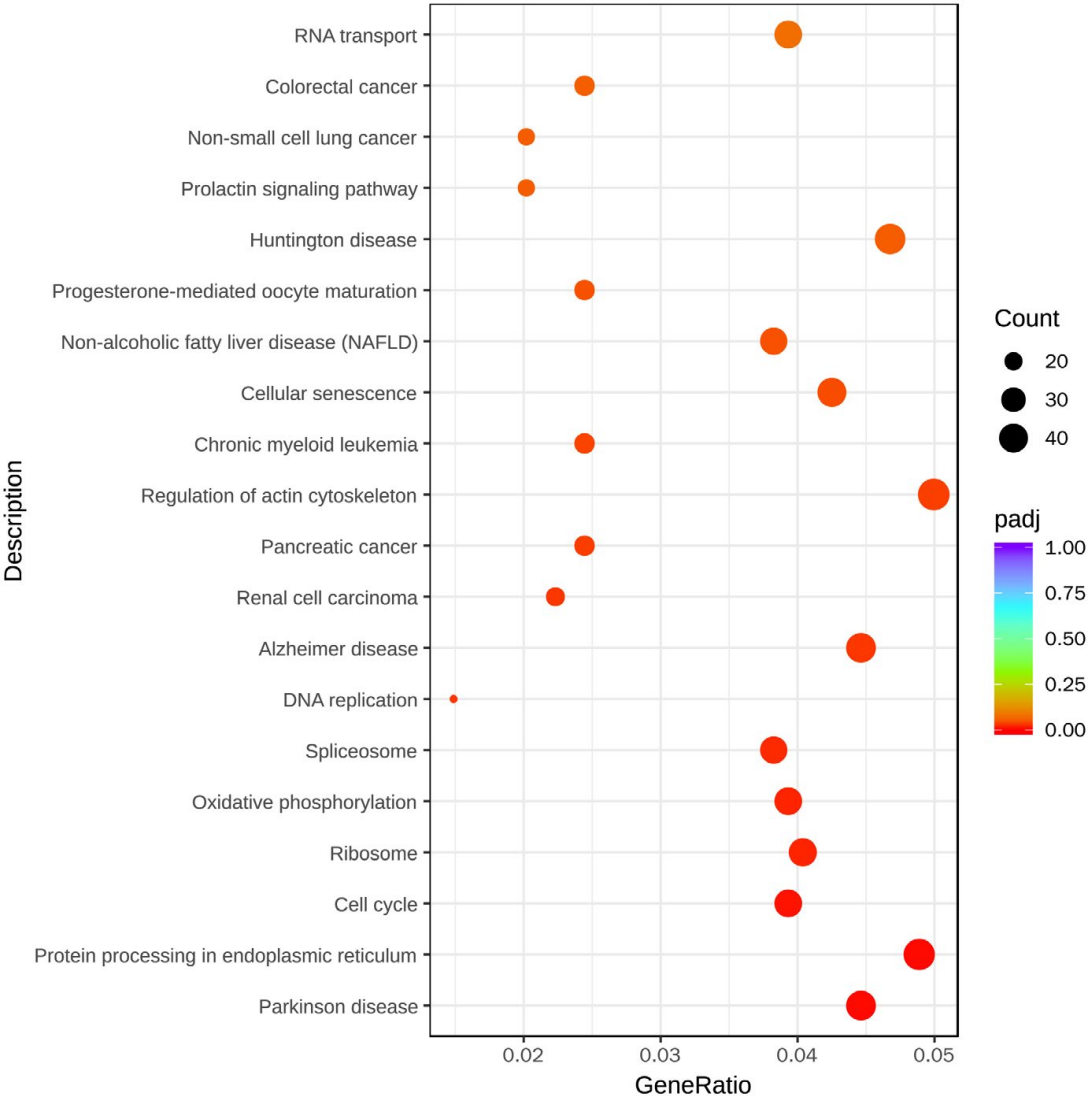
KEGG analysis of Neuro‐2a cells infected by Ad‐Pax3. KEGG pathways analysis revealed 'Parkinson disease', ‘Protein processing in endoplasmic reticulum’ and 'cell cycle' were significant KEGG pathways compared with untreated cells

KEGG database reveals the pathways that might be regulated under Pax3 overexpressing condition for the Neuro‐2a cells. As shown in Figure 7, 'Parkinson disease', 'Protein processing in endoplasmic reticulum' and 'cell cycle' are significant KEGG pathways. Pathway analysis revealed that Pax3 affects cell cycle of Neuro‐2a cell through regulating proproliferative cell cycle genes, such as cell division cycle 6 (CDC6), origin recognition complex submit 4 (ORC4) and minichromosome maintenance complex component 2‐7 (MCM2‐7) (Table S1). Therefore, Pax3 promoted up‐regulation of genes involved in cell cycle machinery, which indicated the potential mechanism of proliferation.

## 4 DISCUSSION

The function of Pax gene family has been widely examined. The roles of Pax3 and Pax7 in the spinal cord development are relatively well understood, but it is still unknown the function of Pax3 and Pax7 in postnatal nervous system development. The Neuro‐2a cell line was originally derived from mouse neuroblastoma cells of a spontaneous tumour. The cells are a type of brain tumour cell, but they carry many inherent morphological and physiological properties that resemble neural stem cells in neuronal development. Pax3 has been widely studied in lots of studies about Pax gene function in the development of neural crest, whereas pax7 is important for the development and the postnatal maintenance of muscle stem cells.^34^ Pax7 mutants are viable until 2‐3 weeks after birth and become more and more severe in the postnatal period when extensive muscle growth takes place.^35^, ^36^

We found that Pax3 not only can inhibit the cell viability and proliferation but also affect the cell cycle and the neurite outgrowth of Neuro‐2a cells. However, there seemed no difference between the Pax7 overexpressed group and the negative control group. Considering the low expression level of Pax7 in Neuro‐2a cells and the role in postnatal nervous system, Pax7 may not play essential roles in Neuro‐2a cells proliferation and differentiation. The effects of Pax3 on cell viability have been checked in other cell lines in which the Pax3 expression had relatively high level. However, no significant change of cell viability was found after Pax3 overexpression in the LLC lung cancer cell line, the 4T1 murine breast cancer cell line and the MC38 colon cancer cell line (Figure S4). It suggested the mechanism of Pax3 on cell viability is not universal among different cell lines. We also examined cleaved caspase 3(CC3), the apoptosis marker, in Pax3 overexpressed group. Pax7 overexpressed group and negative control group. These results indicated that, even though the cell proliferation has been inhibited, cell apoptosis rate was not increased in Pax3 overexpressed group (Figure S5).

Quiescence is important for long‐term maintenance of adult stem cells. Stem cells can maintain tissue homeostasis through new differentiated cell generation. The ability to enter a reversible state of quiescence keeps adult stem cells away from the damage and protects the population from depletion.^37^ The regenerative capacity of resident neural stem cells (NSCs) is important for neurogenesis in mammalian brain.^38^ Similarity, myogenic progenitor cells, also called satellite cells, can drive rapid activation and regeneration in adult muscle. It was implicated that Pax3 and Pax7 played essential roles in maintaining cell survival and promoting proliferation. It has also been reported that these two paralogs can regulate the entry of myogenic progenitor cells into the skeletal muscle programme, not only in the postnatal stage but also in the adult.^39^, ^40^, ^41^ However, in our study, Pax3 can inhibit the cell viability and proliferation of Neuro‐2a cells. KEGG pathways analysis revealed that Pax3 may promote up‐regulation of genes involved in cell cycle machinery, thereby inhibit the proliferation of Neuro‐2a cells. Several proproliferative cell cycle genes are up‐regulated, including cell division cycle 6, 45, 25a, 25b (CDC6, 45, 25a, 25b), origin recognition complex submit 4 (ORC4) and minichromosome maintenance complex component 2‐7 (MCM2‐7). As reported, the ORC and Cdc6 load a Mcm2‐7 double hexamer onto DNA to start DNA replication.^42^, ^43^, ^44^ Up‐regulation of these genes may strengthen the DNA‐binding, thereby arrest the cell cycle at S phase. Besides, KEGG pathways analysis exhibited that CCNB1 was down‐regulated. CCNB1 mainly expressed in G2/M transition of mitotic cell cycle, which was consistent with the fact that Neuro‐2a cells overexpressing Pax3 were arrested at S phase and showed a concomitant decrease in the G2 phase. Rhabdomyosarcoma, a common soft‐tissue malignancy, resulted from overexpression of Pax3/7‐FOXO1 fusion gene.^45^, ^46^, ^47^ Dysregulated myogenesis was observed in Rhabdomyosarcoma.^48^ However, bioinformatics analysis exhibited that CCNB1 was significant up‐regulated in Rhabdomyosarcoma patient,^49^ which was not consistent with the finding in Neuro‐2a cells overexpressing Pax3. It indicated that CCNB1 may not be the direct target of Pax3.

In conclusion, Pax3, but not Pax7 is a critical regulator of cell viability, proliferation, cell cycle and neurite outgrowth in Neuro‐2a cells.

## CONFLICT OF INTEREST

The authors declare that there are no conflict of interests.

## AUTHORS’CONTRIBUTIONS

WZ and JW: Study design. BH, YY and BY: Animal experiments including tissue collection and RNA/protein extraction. BH, ML and XC: Neuro‐2a cell experiments. JW and WZ: Data analysis; writing the paper. XC and BY: Manuscript revision. All authors: Final approval of manuscript.

## Supporting information

Fig S1

Fig S2

Fig S3

Fig S4

Fig S5

Table S1
