# Supplementary material for: Pax3 inhibits Neuro‐2a cells proliferation and neurite outgrowth
Source: J Cell Mol Med. 2020 Dec 17;25(2):1252–62. doi: 10.1111/jcmm.16195 (PMC7812298; doi:10.1111/jcmm.16195)
Supplement: Supplementary file 1 — Fig S1 [file JCMM-25-1252-s001.pdf]

```
1 Pax3-201           1   MTTLAGAVPRMMRPGPGQNYPRSGFPLEVSTPLGQGRVNQLGGVFINGRPLPNHIRHK
2 PAX3-206(PAX3d)    1   MTTLAGAVPRMMRPGPGQNYPRSGFPLEVSTPLGQGRVNQLGGVFINGRPLPNHIRHK
3 Pax3-202           1   MTTLAGAVPRMMRPGPGQNYPRSGFPLEVSTPLGQGRVNQLGGVFINGRPLPNHIRHK
4 PAX3-204(PAX3c)    1   MTTLAGAVPRMMRPGPGQNYPRSGFPLEVSTPLGQGRVNQLGGVFINGRPLPNHIRHK
5 PAX3-205(PAX3e)    1   MTTLAGAVPRMMRPGPGQNYPRSGFPLEVSTPLGQGRVNQLGGVFINGRPLPNHIRHK
6 PAX3-202(PAX3g)    1   MTTLAGAVPRMMRPGPGQNYPRSGFPLEVSTPLGQGRVNQLGGVFINGRPLPNHIRHK
7 PAX3-203(PAX3h)    1   MTTLAGAVPRMMRPGPGQNYPRSGFPLEVSTPLGQGRVNQLGGVFINGRPLPNHIRHK
8 PAX3-201(PAX3b)    1   MTTLAGAVPRMMRPGPGQNYPRSGFPLEVSTPLGQGRVNQLGGVFINGRPLPNHIRHK
9 PAX3-208(PAX3a)    1   MTTLAGAVPRMMRPGPGQNYPRSGFPLEVSTPLGQGRVNQLGGVFINGRPLPNHIRHK

1 Pax3-201           59  IVEMAHHGIRPCVISRQLRVSHGCVSKILCRYQETGSIRPGAIGGSKPKQVTTPDVEK
2 PAX3-206(PAX3d)    59  IVEMAHHGIRPCVISRQLRVSHGCVSKILCRYQETGSIRPGAIGGSKPKQVTTPDVEK
3 Pax3-202           59  IVEMAHHGIRPCVISRQLRVSHGCVSKILCRYQETGSIRPGAIGGSKPKQVTTPDVEK
4 PAX3-204(PAX3c)    59  IVEMAHHGIRPCVISRQLRVSHGCVSKILCRYQETGSIRPGAIGGSKPKQVTTPDVEK
5 PAX3-205(PAX3e)    59  IVEMAHHGIRPCVISRQLRVSHGCVSKILCRYQETGSIRPGAIGGSKPKQVTTPDVEK
6 PAX3-202(PAX3g)    59  IVEMAHHGIRPCVISRQLRVSHGCVSKILCRYQETGSIRPGAIGGSKPKQVTTPDVEK
7 PAX3-203(PAX3h)    59  IVEMAHHGIRPCVISRQLRVSHGCVSKILCRYQETGSIRPGAIGGSKPKQVTTPDVEK
8 PAX3-201(PAX3b)    59  IVEMAHHGIRPCVISRQLRVSHGCVSKILCRYQETGSIRPGAIGGSKPKQVTTPDVEK
9 PAX3-208(PAX3a)    59  IVEMAHHGIRPCVISRQLRVSHGCVSKILCRYQETGSIRPGAIGGSKPKQVTTPDVEK

1 Pax3-201           117 KIEEYKRENPGMFSWEIRDKLLKDAVCDRNTVPSVSSISRILRSKFGKGEEEEADLER
2 PAX3-206(PAX3d)    117 KIEEYKRENPGMFSWEIRDKLLKDAVCDRNTVPSVSSISRILRSKFGKGEEEEADLER
3 Pax3-202           117 KIEEYKRENPGMFSWEIRDKLLKDAVCDRNTVPSVSSISRILRSKFGKGEEEEADLER
4 PAX3-204(PAX3c)    117 KIEEYKRENPGMFSWEIRDKLLKDAVCDRNTVPSVSSISRILRSKFGKGEEEEADLER
5 PAX3-205(PAX3e)    117 KIEEYKRENPGMFSWEIRDKLLKDAVCDRNTVPSVSSISRILRSKFGKGEEEEADLER
6 PAX3-202(PAX3g)    117 KIEEYKRENPGMFSWEIRDKLLKDAVCDRNTVPSVSSISRILRSKFGKGEEEEADLER
7 PAX3-203(PAX3h)    117 KIEEYKRENPGMFSWEIRDKLLKDAVCDRNTVPSVSSISRILRSKFGKGEEEEADLER
8 PAX3-201(PAX3b)    117 KIEEYKRENPGMFSWEIRDKLLKDAVCDRNTVPSVSSISRILRSKFGKGEEEEADLER
9 PAX3-208(PAX3a)    117 KIEEYKRENPGMFSWEIRDKLLKDAVCDRNTVPSVSSISRILRSKFGKGEEEEADLER

1 Pax3-201           175 KEAEESEKKAKHSIDGILSERASAPQSDEGSDIDSEPDLPLKRKQRRSRTTFTAEQLE
2 PAX3-206(PAX3d)    175 KEAEESEKKAKHSIDGILSERASAPQSDEGSDIDSEPDLPLKRKQRRSRTTFTAEQLE
3 Pax3-202           175 KEAEESEKKAKHSIDGILSERASAPQSDEGSDIDSEPDLPLKRKQRRSRTTFTAEQLE
4 PAX3-204(PAX3c)    175 KEAEESEKKAKHSIDGILSERASAPQSDEGSDIDSEPDLPLKRKQRRSRTTFTAEQLE
5 PAX3-205(PAX3e)    175 KEAEESEKKAKHSIDGILSERASAPQSDEGSDIDSEPDLPLKRKQRRSRTTFTAEQLE
6 PAX3-202(PAX3g)    175 KEAEESEKKAKHSIDGILSERASAPQSDEGSDIDSEPDLPLKRKQRRSRTTFTAEQLE
7 PAX3-203(PAX3h)    175 KEAEESEKKAKHSIDGILSERASAPQSDEGSDIDSEPDLPLKRKQRRSRTTFTAEQLE
8 PAX3-201(PAX3b)    175 KEAEESEKKAKHSIDGILSERGKALVSGVSSH..........................
9 PAX3-208(PAX3a)    175 KEAEESEKKAKHSIDGILSERG....................................

1 Pax3-201           233 ELERAFERTHYPDIYTREELAQRAKLTEARVQVWFSNRRARWRKQAGANQLMAFNHLI
2 PAX3-206(PAX3d)    233 ELERAFERTHYPDIYTREELAQRAKLTEARVQVWFSNRRARWRKQAGANQLMAFNHLI
3 Pax3-202           233 ELERAFERTHYPDIYTREELAQRAKLTEARVQVWFSNRRARWRKQAGANQLMAFNHLI
4 PAX3-204(PAX3c)    233 ELERAFERTHYPDIYTREELAQRAKLTEARVQVWFSNRRARWRKQAGANQLMAFNHLI
5 PAX3-205(PAX3e)    233 ELERAFERTHYPDIYTREELAQRAKLTEARVQVWFSNRRARWRKQAGANQLMAFNHLI
6 PAX3-202(PAX3g)    233 ELERAFERTHYPDIYTREELAQRAKLTEARVQVWFSNRRARWRKQAGANQLMAFNHLI
7 PAX3-203(PAX3h)    233 ELERAFERTHYPDIYTREELAQRAKLTEARVQVWFSNRRARWRKQAGANQLMAFNHLI
8 PAX3-201(PAX3b)        ..........................................................
9 PAX3-208(PAX3a)    197 ...............................KRWRLGRRTCWVTWRASAS........
```

```
1 Pax3-201           291  PGGFPPTAMPTLPTYQLSETSYQPTSIPQAVSDPSSTVHRPQPLPPSTVHQSTIPSNA
2 PAX3-206(PAX3d)    291  PGGFPPTAMPTLPTYQLSETSYQPTSIPQAVSDPSSTVHRPQPLPPSTVHQSTIPSNP
3 Pax3-202           291  PGGFPPTAMPTLPTYQLSETSYQPTSIPQAVSDPSSTVHRPQPLPPSTVHQSTIPSNA
4 PAX3-204(PAX3c)    291  PGGFPPTAMPTLPTYQLSETSYQPTSIPQAVSDPSSTVHRPQPLPPSTVHQSTIPSNP
5 PAX3-205(PAX3e)    291  PGGFPPTAMPTLPTYQLSETSYQPTSIPQAVSDPSSTVHRPQPLPPSTVHQSTIPSNP
6 PAX3-202(PAX3g)    291  PGGFPPTAMPTLPTYQLSETSYQPTSIPQAVSDPSSTVHRPQPLPPSTVHQSTIPSNP
7 PAX3-203(PAX3h)    291  PGGFPPTAMPTLPTYQLSETSYQPTSIPQAVSDPSSTVHRPQPLPPSTVHQSTIPSNP
8 PAX3-201(PAX3b)         ..........................................................
9 PAX3-208(PAX3a)         ..........................................................

1 Pax3-201           349  DSSSAYCLPSTRHGFSSYTDSFVPPSGPSNPMNPTIGNGLSPQVMGLLTNHGGVPHQP
2 PAX3-206(PAX3d)    349  DSSSAYCLPSTRHGFSSYTDSFVPPSGPSNPMNPTIGNGLSPQVMGLLTNHGGVPHQP
3 Pax3-202           349  DSSSAYCLPSTRHGFSSYTDSFVPPSGPSNPMNPTIGNGLSPQVMGLLTNHGGVPHQP
4 PAX3-204(PAX3c)    349  DSSSAYCLPSTRHGFSSYTDSFVPPSGPSNPMNPTIGNGLSPQVMGLLTNHGGVPHQP
5 PAX3-205(PAX3e)    349  DSSSAYCLPSTRHGFSSYTDSFVPPSGPSNPMNPTIGNGLSPQVMGLLTNHGGVPHQP
6 PAX3-202(PAX3g)    349  DSSSAYCLPSTRHGFSSYTDSFVPPSGPSNPMNPTIGNGLSPQVPFIISSQISLGFKS
7 PAX3-203(PAX3h)    349  DSSSAYCLPSTRHGFSSYTDSFVPPSGPSNPMNPTIGNGLSPQVPFIISSQISRK...
8 PAX3-201(PAX3b)         ..........................................................
9 PAX3-208(PAX3a)         ..........................................................

1 Pax3-201           407  QTDYALSPLTGGLEPTTTVSASCSQRLEHMKNVDSLPTSQPYCPPTYSTAGYSMDPVT
2 PAX3-206(PAX3d)    407  QTDYALSPLTGGLEPTTTVSASCSQRLDHMKSLDSLPTSQSYCPPTYSTTGYSMDPVT
3 Pax3-202           407  QTDYALSPLTGGLEPTTTVSASCSQRLEHMKNVDSLPTSQPYCPPTYSTAGYSMDPVT
4 PAX3-204(PAX3c)    407  QTDYALSPLTGGLEPTTTVSASCSQRLDHMKSLDSLPTSQSYCPPTYSTTGYSMDPVT
5 PAX3-205(PAX3e)    407  QTDYALSPLTGGLEPTTTVSASCSQRLDHMKSLDSLPTSQSYCPPTYSTTGYSMDPVT
6 PAX3-202(PAX3g)    407  F.........................................................
7 PAX3-203(PAX3h)         ..........................................................
8 PAX3-201(PAX3b)         ..........................................................
9 PAX3-208(PAX3a)         ..........................................................

1 Pax3-201           465  GYQYGQYGQSAFHYLKPDIA.....................
2 PAX3-206(PAX3d)    465  GYQYGQYGQSAFHYLKPDIA.....................
3 Pax3-202           465  GYQYGQYGQSKPWTF..........................
4 PAX3-204(PAX3c)    465  GYQYGQYGQSKPWTF..........................
5 PAX3-205(PAX3e)    465  GYQYGQYGQSAFHYLKPDIAWFQILLNTFDKSSGEEEDLEQ
6 PAX3-202(PAX3g)         .........................................
7 PAX3-203(PAX3h)         .........................................
8 PAX3-201(PAX3b)         .........................................
9 PAX3-208(PAX3a)         .........................................
```
