# Supplementary figures and images for: Pax3 inhibits Neuro‐2a cells proliferation and neurite outgrowth
Source: J Cell Mol Med. 2020 Dec 17;25(2):1252–62. doi: 10.1111/jcmm.16195 (PMC7812298; doi:10.1111/jcmm.16195)

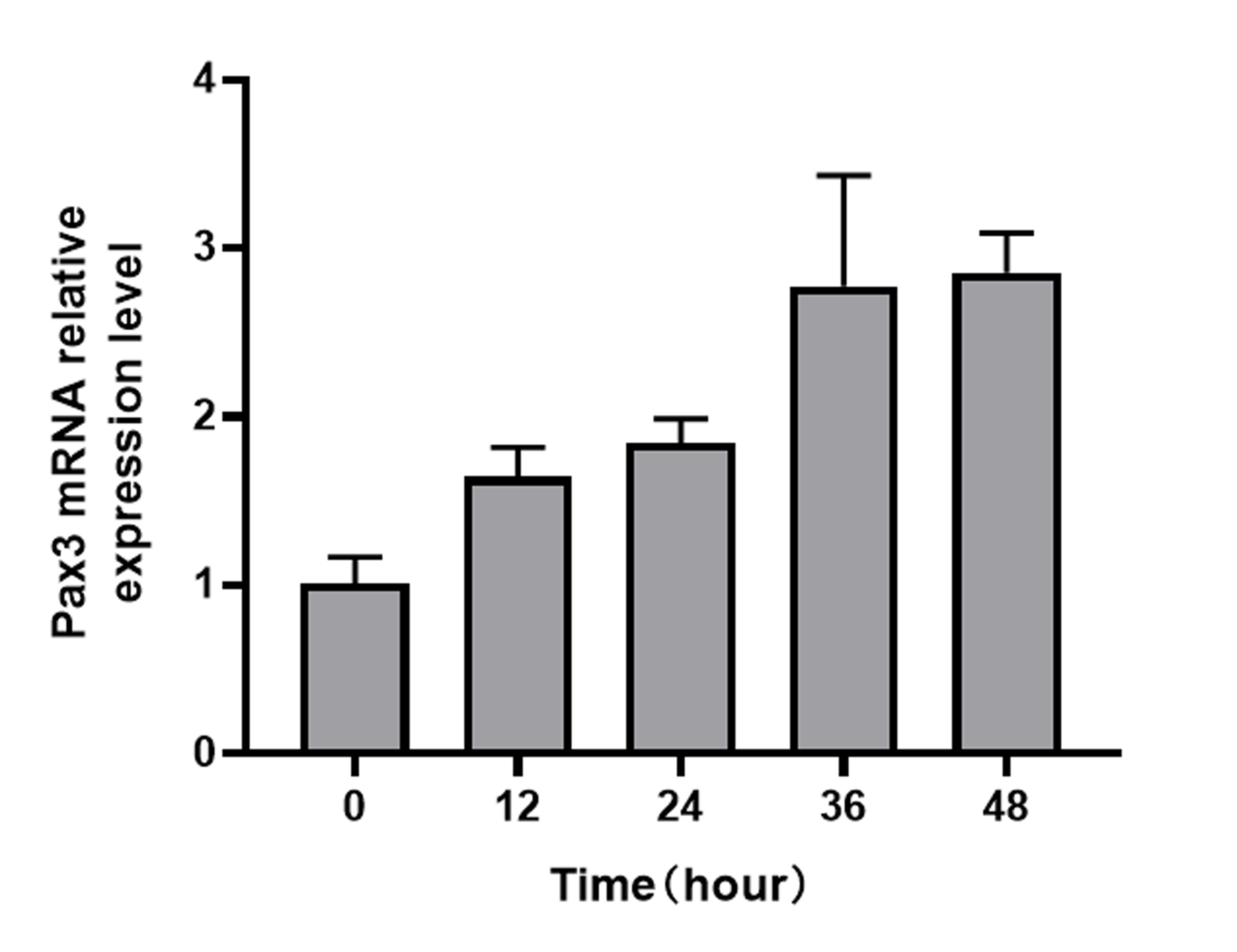

Supplement: Supplementary file 2 — Fig S2 [file JCMM-25-1252-s002.tif]

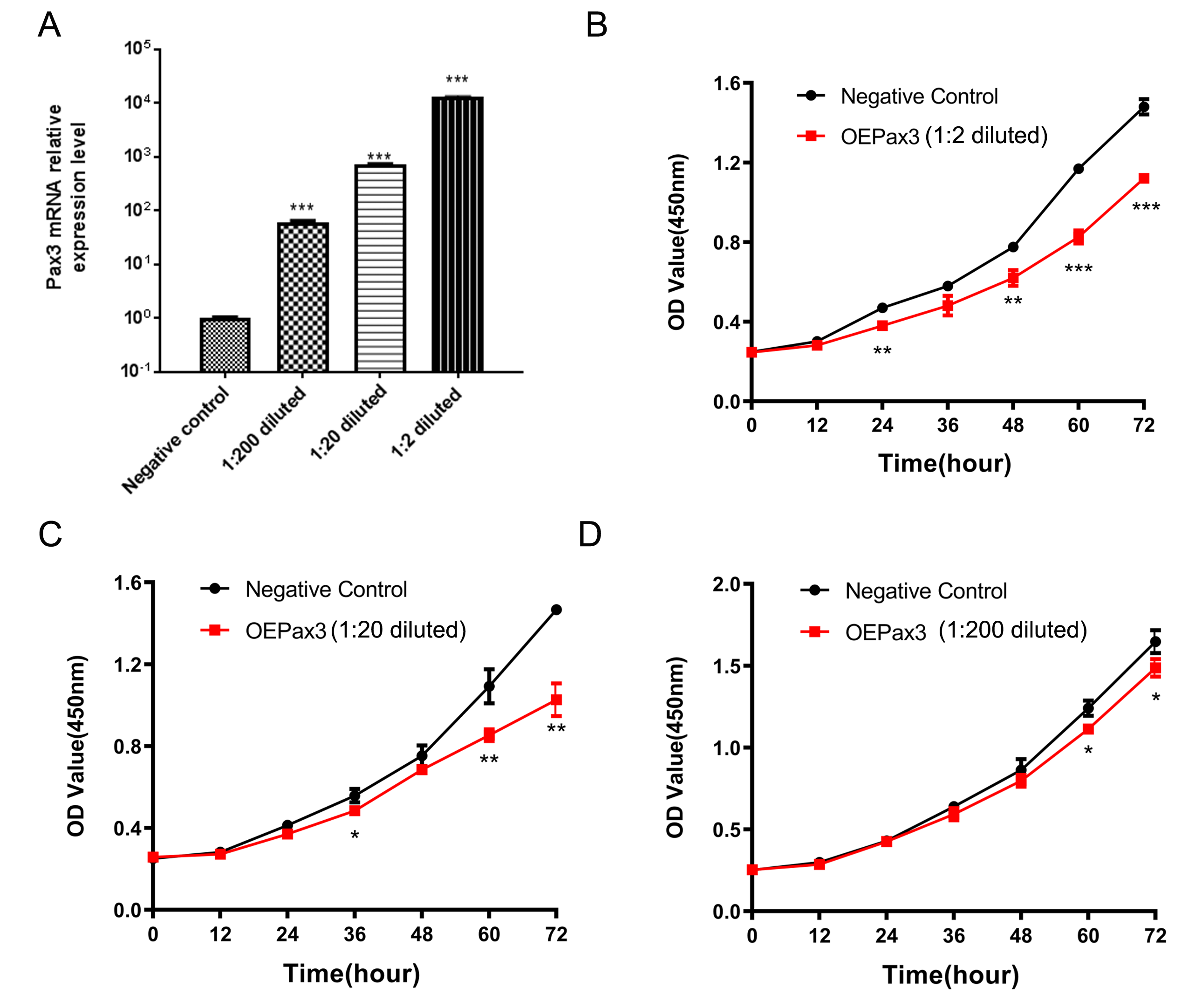

Supplement: Supplementary file 3 — Fig S3 [file JCMM-25-1252-s003.tif]

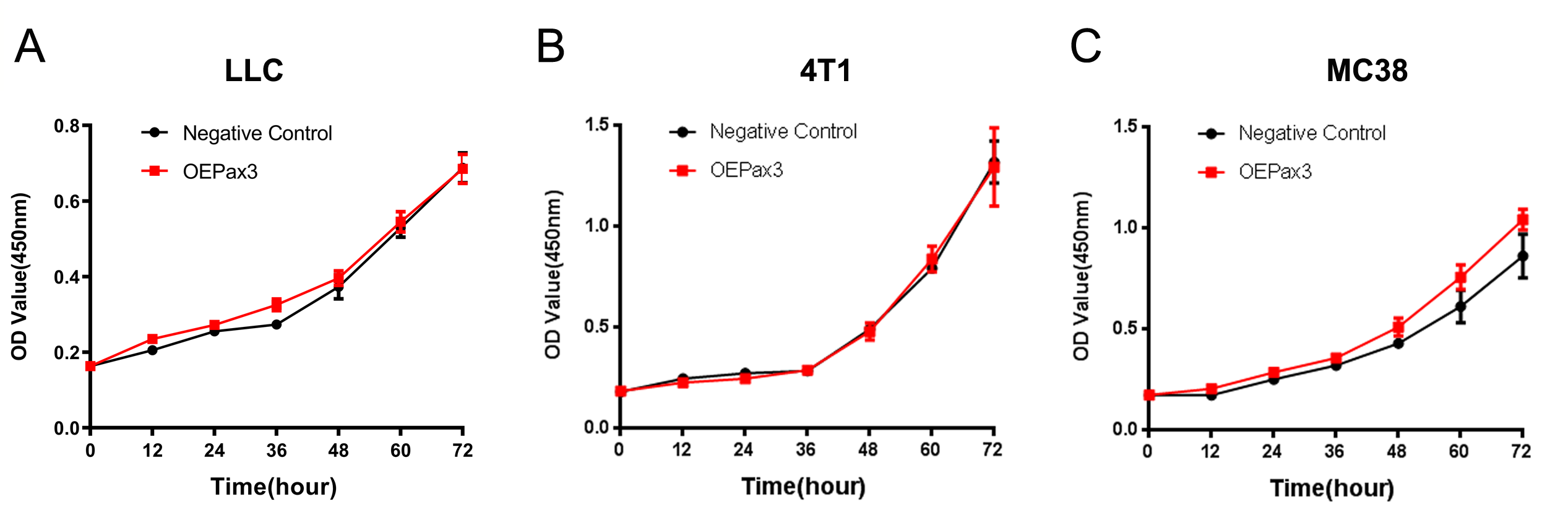

Supplement: Supplementary file 4 — Fig S4 [file JCMM-25-1252-s004.tif]

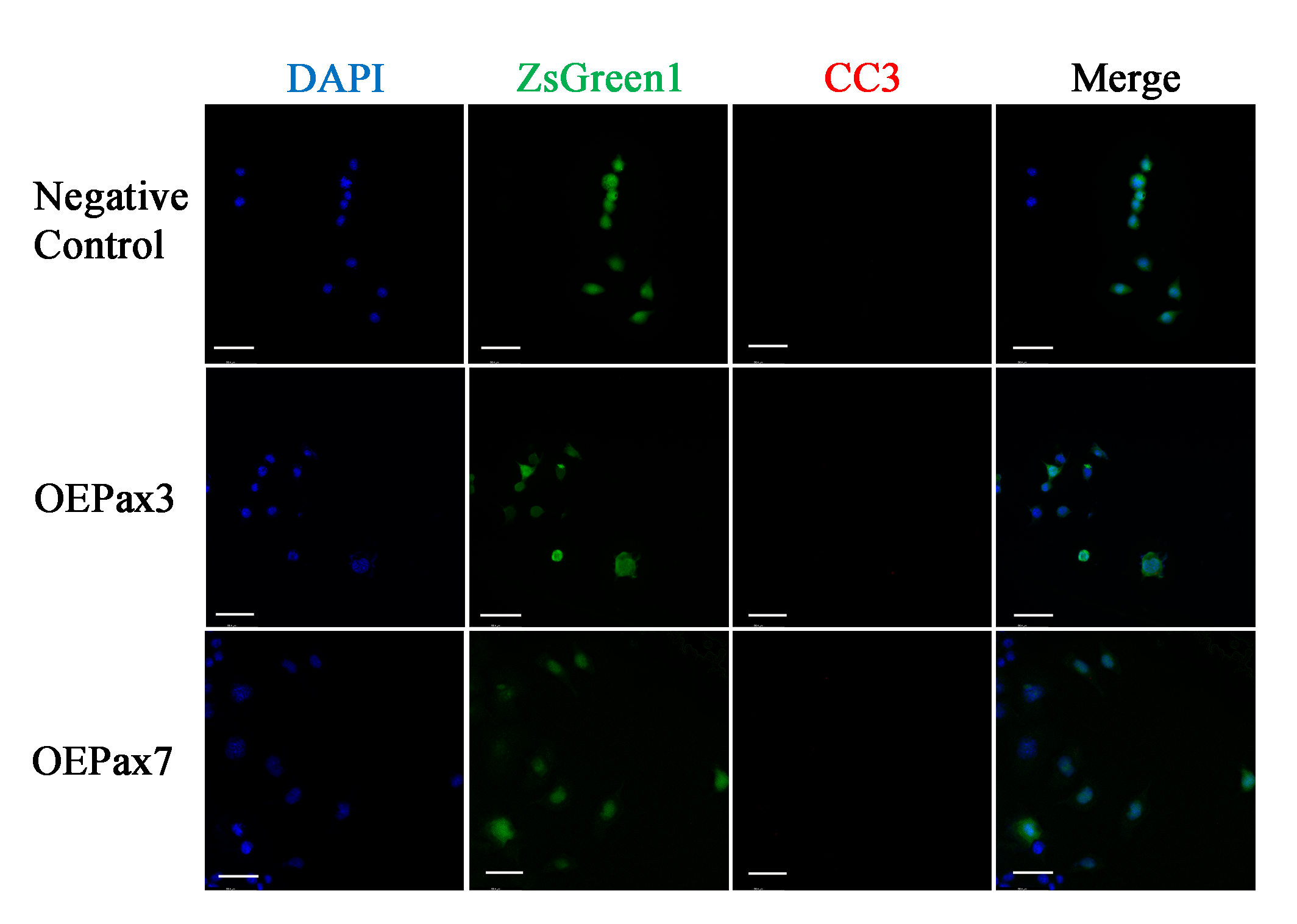

Supplement: Supplementary file 5 — Fig S5 [file JCMM-25-1252-s005.tif]
