# Supplementary material for: Pax3 inhibits Neuro‐2a cells proliferation and neurite outgrowth
Source: J Cell Mol Med. 2020 Dec 17;25(2):1252–62. doi: 10.1111/jcmm.16195 (PMC7812298; doi:10.1111/jcmm.16195)
Supplement: Supplementary file 6 — Table S1 [file JCMM-25-1252-s006.docx]

**Table S1. KEGG analysis of differentially expressed genes from Neuro-2a cells infected by Ad-Pax3.**

| KEGG ID | Description | P value | Gene Name |
| --- | --- | --- | --- |
| mmu05012 | **Parkinson disease** | 6.61E-06 | Atp5b/mt-Nd1/mt-Nd4/mt-Cytb/Cox5b/Slc18a2/Atp5g3/mt-Nd6/Cox4i1/mt-Nd2/Ndufa10/Ndufb8/Prkaca/Atp5g1/Uqcrq/Sdhb/Uba1/Atp5h/Ndufb11/Ndufab1/Atp5d/Ndufa8/Uqcrc1/Uchl1/Atp5a1/Ubb/Cycs/Ube2g2/Cox8a/Ndufb6/Park7/Sdhd/Ndufb10/Atp5c1/Ndufv2/Uqcrh/mt-Co1/Ndufs5/Ndufb7/Ndufa4/Slc18a1/Ndufs4 |
| mmu04141 | **Protein processing in endoplasmic reticulum** | 9.92E-06 | Calr/Ero1l/Hspa4l/P4hb/Rpn1/Hyou1/Hsp90aa1/Rrbp1/Ddost/Hsph1/Lman2/Pdia6/Dnajb1/Sec61a1/Dnajb11/Map3k5/Ern1/Mbtps2/Hsp90ab1/Rnf5/Pdia3/Stub1/Sec61b/Mapk9/Ssr2/Nploc4/Hspa5/Uggt2/Man1c1/Ubqln2/Ube2g2/Dad1/Vcp/Herpud1/Uggt1/Amfr/Mapk8/Sec62/Txndc5/Bag1/Pdia4/Sec23b/Xbp1/Rpn2/Rbx1/Bax |
| mmu04110 | **Cell cycle** | 3.56E-05 | Ccne2/Stag2/Smc3/Bub1/Orc4/Atr/Cdc6/Ccnd1/Mcm2/Cdc27/Hdac1/Ccnd3/Cdc45/Cdc25b/Cdc25a/Ttk/Rb1/Pcna/Mcm4/Mcm7/Mad1l1/Tgfb2/Smad3/Mcm5/Stag1/Mcm6/Tfdp1/Ccnb1/Gadd45b/Orc6/Gsk3b/Ywhah/Espl1/Mcm3/Rbx1/Ccne1/Cdk4 |
| mmu03010 | **Ribosome** | 0.000143 | Rps27a/Rps2/Rps12/Rplp0/Rpl17/Gm11808/Rpl10/Rpl7a/Rpl27/Rps19/Rps3a1/Rpl12/Mrpl12/Rplp1/Rps16/Rpl23/Rpl37a/Mrpl20/Rps25/Rps9/Rpsa/Rpl9/Rps13/Rpl10a/Rps27l/Rps20/Mrpl2/Rpl18/Rps3/Mrpl28/Rpl34/Rpl37/Rpl35/Rps23/Rps17/Rpl18a/Mrpl13/Mrps11 |
| mmu00190 | **Oxidative phosphorylation** | 0.000159 | Atp5b/mt-Nd1/mt-Nd4/mt-Cytb/Cox5b/Atp5g3/mt-Nd6/Cox4i1/mt-Nd2/Ndufa10/Ndufb8/Atp5g1/Uqcrq/Sdhb/Atp5h/Ndufb11/Ndufab1/Atp5d/Ndufa8/Uqcrc1/Atp6v0b/Atp6v1f/Ppa1/Atp5a1/Atp6v0d1/Cox8a/Ndufb6/Sdhd/Ndufb10/Atp5c1/Ndufv2/Uqcrh/mt-Co1/Ndufs5/Ndufb7/Ndufa4/Ndufs4 |
| mmu03040 | **Spliceosome** | 0.000252 | Thoc2/Rbm25/U2surp/Snrpb/Alyref/Lsm4/Sf3a2/Eif4a3/Srsf10/Prpf19/Sf3b1/Srsf3/Syf2/Snrpf/Phf5a/Prpf38b/Snrpa/Hnrnpa3/Dhx15/Snrpd3/Ddx46/Snrpc/Plrg1/Hnrnpc/Thoc1/Cdc40/Sf3b4/Ctnnbl1/Srsf9/Bud31/Thoc3/Rbmxl1/Sf3b2/Eftud2/Lsm7/Sf3b6 |
| mmu03030 | **DNA replication** | 0.000536 | Rpa1/Mcm2/Rpa2/Pcna/Mcm4/Mcm7/Prim1/Mcm5/Mcm6/Pola1/Rfc5/Rnaseh2a/Rpa3/Mcm3 |
| mmu05010 | **Alzheimer disease** | 0.000568 | Gapdh/Mapt/Atp5b/mt-Cytb/Cox5b/Atp5g3/Cox4i1/Ndufa10/Ndufb8/Psen2/Atp5g1/Ern1/Uqcrq/Sdhb/Atp5h/Ndufb11/Ndufab1/Plcb4/Atp5d/Ndufa8/Uqcrc1/Atp5a1/Cycs/Cox8a/Ndufb6/Mapk1/Sdhd/Ndufb10/Gsk3b/Tnfrsf1a/Atp5c1/Ndufv2/Uqcrh/mt-Co1/Ppp3cb/Ndufs5/Ndufb7/Calm3/Ndufa4/Atp2a3/Ndufs4/Gnaq |
| mmu05211 | **Renal cell carcinoma** | 0.000612 | Vegfa/Rap1a/Hif1a/Braf/Elob/Tgfb2/Grb2/Crk/Map2k2/Arnt/Pik3r1/Kras/Ets1/Mapk1/Akt1/Pik3r2/Pak4/Sos2/Fh1/Vhl/Rbx1 |
| mmu05212 | **Pancreatic cancer** | 0.000813 | Brca2/Tgfbr1/Ccnd1/Vegfa/Braf/Rad51/Rb1/Tgfb2/Smad3/Jak1/Polk/Mapk9/Pik3r1/Ralgds/Gadd45b/Bcl2l1/Kras/Mapk8/Mapk1/Akt1/Pik3r2/Bax/Cdk4 |
| mmu04810 | **Regulation of actin cytoskeleton** | 0.000974 | Rock2/Rock1/Itga6/Itga2/Gna13/Apc/F2r/Msn/Nckap1/Braf/Fgf1/Actn1/Pdgfra/Diaph3/Ppp1cb/Arpc5l/Itgb1/Ppp1ca/Pfn1/Myh10/Crk/Map2k2/Pip4k2c/Iqgap2/Itga1/Itgav/Itga7/Pik3r1/Kras/Pikfyve/Bdkrb2/Cfl2/Mapk1/Ssh2/Pik3r2/Chrm1/Pak4/Limk1/Sos2/Actn4/Ezr/Fgd1/Ppp1r12a/Myh9/Fgf6/Cfl1/Cyfip2 |
| mmu05220 | **Chronic myeloid leukemia** | 0.001222 | Tgfbr1/Ccnd1/Stat5b/Hdac1/Braf/Rb1/Tgfb2/Smad3/Polk/Grb2/Crk/Map2k2/Nfkbia/Pik3r1/Gadd45b/Bcl2l1/Kras/Mapk1/Akt1/Pik3r2/Sos2/Bax/Cdk4 |
| mmu04218 | **Cellular senescence** | 0.001618 | Ccne2/Atr/Trpm7/Tgfbr1/Mapk14/Ccnd1/Sqstm1/Ccnd3/Foxo3/Cdc25a/Rb1/Ppp1cb/H2-K1/Tgfb2/Smad3/Ppp1ca/Rbbp4/Ppid/Zfp36l2/Map2k2/Foxm1/Ccnb1/Pik3r1/H2-D1/Gadd45b/Il6/Kras/Ets1/Lin54/Tsc2/Mapk1/Akt1/Pik3r2/Hipk1/H2-Q1/Ppp3cb/Calm3/Ccne1/Mybl2/Cdk4 |
| mmu04932 | **Non-alcoholic fatty liver disease (NAFLD)** | 0.002064 | Irs2/mt-Cytb/Cox5b/Cox4i1/Ndufa10/Ndufb8/Map3k5/Ern1/Uqcrq/Sdhb/Ndufb11/Ndufab1/Mapk9/Ndufa8/Uqcrc1/Pik3r1/Cycs/Il6/Cox8a/Ndufb6/Mapk8/Sdhd/Akt1/Ndufb10/Gsk3b/Pik3r2/Tnfrsf1a/Ndufv2/Uqcrh/mt-Co1/Ndufs5/Ndufb7/Xbp1/Ndufa4/Bax/Ndufs4 |
| mmu04914 | **Progesterone-mediated oocyte maturation** | 0.002163 | Bub1/Mapk14/Rps6ka3/Cdc27/Hsp90aa1/Cpeb4/Cpeb2/Cdc25b/Braf/Cdc25a/Prkaca/Mad1l1/Rps6ka2/Hsp90ab1/Mapk9/Ccnb1/Pik3r1/Kras/Mapk8/Mapk1/Akt1/Pik3r2/Spdye4a |
